# The therapeutic potential of CRTH2/DP2 beyond allergy and asthma

**DOI:** 10.1016/j.prostaglandins.2017.08.006

**Published:** 2017-08-14

**Authors:** Katharina Jandl, Akos Heinemann

**Affiliations:** a Institute for Experimental and Clinical Pharmacology, Medical University Graz, Austria; b Ludwig Boltzmann Institute for Lung Vascular Research, Graz, Austria; c BioTechMed Graz, Austria

**Keywords:** CRTH2, DP2, DP1, PGD_2_, Inflammation pharmacological target

## Abstract

Prostaglandin (PG) D_2_ has been in the focus of research for quite a long time, but its biological effects and its roles in human disease are still not fully characterized. When in 2001 a second major PGD_2_ receptor termed chemoattractant receptor homologue expressed on Th2 cells (CRTH2; alternative name DP2) was discovered, diverse investigations started to shed more light on the complex and often controversial actions of the prostaglandin. With various immunomodulating effects, such as induction of migration, activation, and cytokine release of leukocytes observed both in vivo and in vitro, CRTH2 has emerged as a promising target for the treatment of allergic diseases. However, with more and more research being performed on CRTH2, it has also become clear that its biological actions are far more diverse than expected at the beginning. In this review, we aim to summarize the roles that PGD_2_ – and CRTH2 in particular – might play in diseases of the central nervous system, kidney, intestine, lung, hair and skin, bone and cartilage, and in cancer. Based on current data we propose that blocking CRTH2 might be a potential therapeutic approach to numerous conditions beyond classical allergic diseases and asthma.

## 1 CRTH2 – history and clinical potential

Among prostaglandins (PG), PGD2 remained the most elusive species for a long time and was initially regarded as having negligible biological activity [1]. In 1974 its inhibitory effect on platelet aggregation was discovered by Smith et al. [2] and Mills & McFarlain [3], and both pressor and depressor actions were found in different smooth muscle preparations by Horton et al. [4]. In 1976, pro-inflammatory actions of PGD_2_ were described by Flower et al. in rat and human skin, causing erythema and edema, however, in the absence of pain [5]. In dog lung, PGD2 was observed to cause broncho- and vasoconstriction, while causing systemic hypotension [6] and renal vasodilation [7]. In contrast, guinea pig coronary arteries were constricted by PGD2 [8,9]. Later it was shown that it was the thromboxane receptor, TP, that mediated these constrictor effects, as PGD2 was found also to bind to TP at micromolar concentrations [10], whereas inhibition of platelet aggregation and vasodilation by PGD_2_ depended on its cognate D-type prostaglandin receptor, DP (also named DP1) [11]. In 1978, Anhut et al. [12] suggested that PGD_2_ was formed during anaphylactic reactions, which might contribute to broncho- and vasoconstriction during asthma attacks, as they hypothesized. Four years later, Lewis et al. demonstrated that mast cells were a major source of PGD_2_ [13]. Although Peskar & Brune already proposed in 1979 that PGD2 was the prevailing PG in acute inflammatory responses [14], its immune modulator mode of action still needed to be elucidated. In dogs, two studies indirectly suggested that PGD2 might be a chemoattractant for eosinophils, the first showing that intravenous PGD_2_ caused a transient drop in circulating eosinophil numbers [15], and the second that intratracheal PGD2 caused intra-luminal eosinophil accumulation [16]. In 1990, Woodward et al. described the ocular hypotensive effect of PGD_2_ and the selective DP1 agonist BW245c in guinea pigs [17]. However, they also found that PGD_2_–but not the DP1 agonist – induced ocular inflammation characterized by accumulation of eosinophils in the conjunctiva. Interestingly, the PGD2 metabolite PGJ2 was as effective as PGD_2_ in causing eosinophil accumulation, but was unable to decrease ocular pressure, which pointed to a yet unknown PGD2 receptor. Subsequently, PGD_2_ was shown to stimulate the migration of eosinophils towards zymosan-activated serum and induce calcium flux in human eosinophils [18,19], but it was only in 2001 that PGD_2_ was unequivocally shown to be a potent eosinophil chemoattractant acting through a novel receptor termed chemoattractant receptor homologue expressed on Th2 cells (CRTH2; alternative name: DP2) [20–22]. This receptor had previously been cloned as an orphan receptor (GPR44) that was expressed by eosinophils, basophils and Th2 lymphocytes [23]. In fact, CRTH2 was characterized as the most reliable surface marker for Th2 cells [24]. With these findings in mind, PGD2 and its receptor CRTH2 has become one of the most promising therapeutic targets in the field of allergy and asthma, which was also fueled by the discovery of indomethacin as a potent and selective CRTH2 agonist. This clinically used cyclooxygenase inhibitor subsequently served as a pharmacophore for the development of several CRTH2 antagonists [25], belonging to the family of indole-acetic acid derivatives. Some of those including OC000459, or AZD1981 have already been evaluated in clinical studies for the treatment of asthma, allergic rhinitis and eosinophilic esophagitis [26–30]. Although, major breakthroughs in the clinical usefulness of CRTH2 antagonists are still to be anticipated, recent studies in allergic asthma are showing promising results: Fevipriprant improved lung function in a subgroup of patients suffering from severe air flow limitation [31] and timapriprant (OC000459) beneficially altered asthma control as well as lung function in atopic eosinophilic asthmatics [32]. Timapiprant and another CRTH2 antagonist, BI 671800, also successfully reduced nasal and ocular symptoms in allergic subjects exposed to grass pollen [27,33]. For a detailed review of PGD2 receptor antagonists in the treatment of asthma, please refer to the recent review by Santus and Radovanovic [25].

Ironically, the purported TP antagonist ramatroban (BAY u 3405) which had already been marketed in Japan as a treatment of allergic rhinitis, was also revealed to be a potent CRTH2 antagonist [34].

## 2 CRTH2 beyond allergy and asthma

Meanwhile, CRTH2 has been found to be expressed on several additional cell types and in different tissues suggesting that the PGD_2_/CRTH2 axis might be of potential relevance beyond allergy and asthma. Although the role of PGD_2_ in a Th2-biased inflammation is well established, investigation of its function in other groups of inflammatory reactions in experimental mouse models is confounded by differential expression patterns of CRTH2 in mice and humans: While CRTH2 can be used as an exclusive marker for Th2 cells in humans, CRTH2-positive Th1 cells as well as neutrophils are present in mice. These differences have to be taken into consideration when drawing conclusions from studies exclusively based on mouse data. A detailed summary of the presence or absence of CRTH2 on various cells types can be found in Table 1.

### 2.1 Respiratory tract

In the human lung, the majority of structural (epithelium and endothelium) and immune cells (including macrophages, monocytes, mast cells, Th2 cells and eosinophils) express CRTH2 receptors. Interestingly, CRTH2 expression levels as well as the ratio of CRTH2-positive vs CRTH2-negative cells have been reported to correlate with disease activity. In scleroderma, an increased ratio of CCR5- vs CRTH2-expressing cells in the circulating T lymphocyte population was associated with a persistent involvement of the lung vasculature manifested as pulmonary arterial hypertension. This state of a high CCR5/CRTH2 ratio was associated with a poorer prognosis and a profibrotic phenotype in scleroderma patients [51]. In experimental fibrosis induced by bleomycin application, hematopoietic PGD synthase-deficient mice exhibited a more severe phenotype. Although the authors did not assess the specific receptors involved, a protective role of PGD_2_ in fibrosis was proposed [52]. It is reasonable to at least partially attest the protective role of PGD_2_ in pulmonary fibrosis to both direct anti-proliferative effect on fibroblasts [53] and anti-fibrotic effects mediated by inhibition of TGF-beta-induced collagen production by DP1 receptor activation [54]. In addition, an involvement of CRTH2 receptors seems also likely, as earlier studies using indomethacin found reduced collagen content and improved lung histopathology after intratracheal administration of bleomycin (primarily inducing lung damage and fibrosis) [55] as well as after systemic bleomycin administration (causing multiple organ fibrosis) [56]. In support of these findings, preliminary reports also suggested that bleomycyin-induced pulmonary fibrosis was aggravated in CRTH2-knockout mice, displaying higher mortality rate, reduced pulmonary compliance and increased inflammation and collagen deposition [57,58]. This notion of an anti-fibrotic action of PGD2 was further substantiated by the ability of CRTH2/PGD2 to inhibit epithelial-to-mesenchymal transition, a process observed during development of fibrosis [59]. Unfortunately, the involvement of CRTH2 receptors has not been assessed in human pulmonary fibrosis thus far. Given the differential expression of CRTH2 receptors in mice and humans, the antifibrotic effects in experimental fibrosis may not be directly transferable to human disease. Indeed, at variance with the murine studies, Zhou and colleagues described a profibrotic role of CRTH2 in the inherited disorder Hermansky-Pudlak syndrome. This disease can present with pulmonary fibrosis as a leading cause of mortality. The authors here described a functional interaction of CRTH2 and chitinase 3-like-1 (CHI3L1) resulting in increased pro-fibrotic signaling [60]. Hence, these data suggest that CRTH2 can be associated both with anti- and pro-fibrotic events.

CRTH2 has further been found to contribute to acute lung inflammation. In a murine model of endotoxin-induced acute lung injury,we found that CRTH2 activation led to an early-phase polarization of alveolar macrophages resulting in a lung milieu favoring neutrophil recruitment and, therefore, inducing a more severe phenotype with regard to lung histo-pathology as well as lung function. In this study, activation of CRTH2 on macrophages induced a pro-inflammatory phenotype leading to elevated levels of proinflammatory cytokines, such as tumor necrosis factor-alpha (TNF-α), monocyte chemotactic protein-1 and keratinocyte-derived cytokine in the bronchoalveolar lavage fluid (BALF) which – in turn – stimulated neutrophils. Accordingly, a measurable increase in endogenous PGD2 levels was detected in the BALF of endotoxin-treated animals and pharmacological blockade of CRTH2 ameliorated alveolar neutrophil influx into the lungs. Although murine neutrophils are known to express CRTH2 receptors (as mentioned in the previous section), then pro-inflammatory actions on neutrophils were not due to direct activation by PGD_2_ but via macrophage activation. [40]. In a more severe form of LPS-induced acute lung injury, PGD_2_ was found to play a protective role, which seemed to depend on the DP1 receptor rather than CRTH2 [37]. Therefore, in acute inflammation, CRTH2 activation is likely to induce a proinflammatory signature in the lung.

The prominent role of CRTH2 in the lung prompted investigations to evaluate the potential of the CRTH2 antagonist AZ11805131 in tobacco smoke-induced airway inflammation, modelling chronic obstructive pulmonary disease (COPD) [61]. The decreased levels of bronchoalveolar lavage neutrophils, macrophages and lymphocytes as well as an improved lung mucosal pathology upon CRTH2 antagonism showed promising results in this mouse study. In the same year another study provided further support for the therapeutic potential of CRTH2 antagonism in both acute as well as sub-chronic murine models of cigarette-induced airway inflammation. In this study, the potent CRTH2 antagonists AM156 and AM206 inhibited neutrophil and lymphocyte recruitment, and additionally also ameliorated airway inflammation by reduction of airway epithelial thickening and mucus cell metaplasia [62]. With these promising results from murine experimental studies, the CRTH2 antagonist AZD1981 was tested in COPD patients. Unfortunately, the positive effects observed in murine models were not replicated in a phase II trial of the CRTH2 antagonist in COPD patients [63]. In consideration that COPD patients present with a Th1 skewing, it cannot be excluded that the beneficial effect in the murine models resulted from an antagonistic action on Th1 cells and neutrophils, which might not be the case in human pathology.

### 2.2 Kidney

In the renal system, increased expression of the lipocalin-like PGD synthase (L-PGDS) has been reported in early stage diabetic nephropathy in rats [64] and adriamycin-induced nephropathy in mice [65], suggesting a possible contribution of PGD2 in chronic kidney disease. To our knowledge, so far only one study investigated the functional role of CRTH2-mediated PGD_2_ effects in kidney disease. Here, the authors corroborated the previous findings of increased L-PGDS expression in another model of chronic kidney disease induced by ureteral obstruction. Furthermore, genetic as well as pharmacological blockade of CRTH2 signaling strongly reduced renal fibrosis and inflammation via suppression of the interleukin (IL)-4/IL-13 axis[66]. Hence, there is a clear involvement of PGD_2_ in the renal system, with elevated levels of PGD_2_ after induction of various forms of kidney pathology, and a profibrotic role of CRTH2 activation. Therefore, although data in humans are still lacking, CRTH2 antagonists might also be a promising approach to kidney disease.

### 2.3 Gastrointestinal tract

Increasing evidence further suggests that CRTH2 might evolve as a promising therapeutic target in inflammatory bowel diseases. In patients suffering from Crohn’s disease, which can affect the entire gastrointestinal tract, we found increased serum levels of PGD_2_ and its metabolite Δ(12)-PGJ_2_, and in a corresponding mouse model of colitis induced by 2,4,6-trinitrobenzenesulfonic acid, the CRTH2 antagonist timapiprant ameliorated inflammation via inhibition of pro-inflammatory mediators TNF-α, IL-1β and IL-6 [67]. In ulcerative colitis, where inflammatory reactions are limited to the colon, we investigated CRTH2 expression in peripheral blood cells and observed an inverse correlation of CRTH2 expression on peripheral blood eosinophils and disease activity in affected patients. We also found that CRTH2 antagonism in a murine model of dextran sulfate sodium-induced colitis improved disease activity with regard to inflammation score, myeloperoxidase levels and weight loss [68]. Previously it was noted that the numbers of CRTH2-positive cells, most likely CD4-positive lymphocytes, were increased in mildly inflamed mucosa and at the margins of more severely inflamed areas in patients with ulcerative colitis [69]. These findings suggest that both in mice and humans the involvement of a Th2-dominated immune response may be possible in the early pathogenesis of inflammatory bowel disease. Peripheral blood eosinophils of patients with eosinophilic esophagitis showed enhanced CRTH2 expression, among other markers [70,71]. Supporting this pro-inflammatory role of CRTH2 in IBD, timapiprant significantly reduced eosinophil infiltration in the tissue and induced some clinical improvement in eosinophilic esophagitis patients [28].

### 2.4 Bone and cartilage

Interestingly, PGD2 can potently modulate bone metabolism with its capacity to induce collagen synthesis during the process of calcification [72] and IL-6 secretion by osteoblasts [73]. In 2005, Gallant and colleagues described both the production of PGD_2_ by, and the presence of both DP1 and CRTH2 receptors on, human osteoblasts. Selective CRTH2 activation in osteoblasts resulted in an increased production of osteoprotegerin, suggesting an autocrine and/or paracrine function of the PGD2-CRTH2 axis in bone anabolism [74]. In human differentiated osteoclasts, CRTH2 stimulation induced lamellipodia formation via actin reorganization, a process crucial for motility and bone resorption. Consequently, CRTH2 antagonism inhibited vitamin D3-induced bone resorption and osteoclastogenesis [75]. In addition, CRTH2 has been proposed as an inducer of apoptosis in osteoclasts via the intrinsic pathway, depending on caspase 9 activity [76] as a consequence of Erk1/2 and Akt signaling [77]. Osteoclast activation also plays a role in arthritis. Interestingly, a murine model of adjuvant-induced joint inflammation revealed that CRTH2-deficient mice develop a more severe phenotype with increased levels of paw swelling and infiltration of inflammatory cells, particularly CD68+ macrophages, which appeared to accelerate the inflammatory response [78]. Noteworthy, this model does not involve T-cell infiltration in the affected joints, which is a clear limitation when compared to the adaptive autoimmune response observed in human rheumatoid arthritis. Interestingly, treatment of mice with a CRTH2 antagonist did not modify disease severity in a different experimental model of rheumatoid arthritis, i.e. collagen-induced arthritis, while selective activation of DP1 proved beneficial [79]. Together, the role of PGD_2_ receptors in bone disorders and arthritis still needs to be clarified.

### 2.5 Nervous system

Inflammation, especially if inappropriately controlled, cannot only lead to a chronic state, but also induce signaling pathways in the brain that influence behavior, emotion and cognitive function. PGD_2_ signaling via DP1 is known to regulate crucial CNS-related functions such as food intake [80] and the sleep–wake cycle [81,82]. Increasing numbers of studies are now also addressing a link between PGD_2_ and CRTH2, and the modulation of cognitive function. A role for the PGD_2_ metabolite, 15-deoxy-PGJ_2_ in the central nervous system was first described in 1999 as an enhancer of nerve growth factor-mediated neurite outgrowth, a function which appeared to be independent from PPARγ and DP1 receptors [83,84], but involved CRTH2 receptors [85]. Additionally, PGD_2_ produced by astrocytes carrying the amyotrophic lateral sclerosis-causing gene SOD1 was identified to contribute to the devastating process of motor-neuron degeneration conferred by glial cells, but blockade of the DP1 receptor only slightly reversed motor neuron loss, tentatively suggesting a potential role for CRTH2 [86–88]. In the peripheral nervous system the PGD2eCRTH2 axis contributes to myelination, as both genetic deletion and pharmacological inhibition of L-PGDS as well as genetic ablation of CRTH2/DP2 caused myelin damage and hypomyelination [89].

A direct link between CRTH2 activation and cognitive dysfunction was proposed recently in mice. LPS-induced sickness behavior, social impairment as well as induction of c-Fos expression in the hypothalamic paraventricular nucleus and central amygdala were dependent on the presence of CRTH2 and were reversed by CRTH2 antagonists. Similar effects were observed with regards to social impairment after tumor inoculation [90]. In a model of cognitive dysfunction induced by the N-methyl-D-aspartate receptor antagonist, MK-801, both pharmacological inhibition and genetic deletion of CRTH2 were shown to be beneficial [91]. Thus, while CRTH2 is essential/involved in myelination and neurite outgrowth, it might also contribute to sickness-induced changes in cognitive function and behavior.

### 2.6 Skin

Prostaglandins have long been implicated in skin homeostasis [92]. Human and mouse keratinocytes produce PGD_2_ and express both PGD_2_ receptors [93,94]. Stimulation of CRTH2 leads to release of the anti-microbial factor beta-defensin-3 from human keratinocytes [93], suggesting a protective effect of the prostaglandin. However, several mouse models have shown that PGD_2_ and it receptor CRTH2 are actively involved in allergic skin inflammation [95,50,96–100]. Moreover, peripheral blood eosinophils and CD4-positive T cells of patients with allergic skin disease have been shown to express higher levels of CRTH2 as compared to healthy controls [101,102]. In a model of chronic skin inflammation, transgenic mice overexpressing lypocalin-type PGD synthase exhibited a complex phenotype: While PGD_2_ acting via DP1 ameliorated the early phase of croton oil-induced skin-inflammation due to its barrier-enhancing properties, PGD_2_ acting via CRTH2 prolonged and worsened the later phase of the inflammatory response by promoting neutrophil activation [103]. CRTH2 seemed to outweigh DP1-mediated responses which – in this specific model – resulted in an overall exaggerated inflammatory response mediated by CRTH2.

CRTH2 might also play a role in eosinophilic pustular folliculitis, which is a chronic pruritic skin disease characterized by massive eosinophil infiltrates of sebaceous glands. One treatment option for the disease is systemic administration of the COX inhibitor and CRTH2 agonist, indomethacin. In addition to abrogating prostaglandin synthesis, indomethacin was found to reduce CRTH2 expression in peripheral blood eosinophils and lymphocytes, probably thereby preventing their recruitment to inflamed skin [104,105].

Bimatoprost, a PGF2α analogue used to decrease ocular pressure in glaucoma, stimulates the growth of eyelash hair as a side effect [106]. In 2012, PGD_2_ and lipocalin-type PGD synthase were found at abundant levels in male scalp tissue of balding areas as compared to non-balding areas [107]. The authors found a direct inhibitory effect of PGD_2_ on hair growth that could be attributed to its action on CRTH2. Moreover, PGD_2_ inhibited hair follicle regeneration in a mouse model of dermal injury in a CRTH2-dependent manner [108]. Previously both DP1 and CRTH2 were found to be present in hair follicles [109]. Another study described that 15-deoxy-PGJ_2_ induces keratinocyte apoptosis, thereby contributing to PGD_2_-induced inhibition of hair growth [110]. Setipiprant, an orally available CRTH2 antagonist, is purportedly investigated in the treatment of androgenic alopecia in a phase II study.

### 2.7 CRTH2 in cancer

Inflammation is a two-edged sword, on the one hand fighting pathogens to limit tissue damage and promote healing, but if inappropriate in nature and degree on the other hand, inflammation itself can drive tissue damage. This is not only the case in allergy and autoimmune disorders but also in cancer [111]. It is now well established that there is both cancer-related inflammation as well as inflammation-induced cancer [112,113]. This interaction is established both via direct cell-to-cell interaction as well as communication by inflammatory mediators such as cytokines or prostanoids. Some of the pro-apoptotic properties of PGD2 and its metabolites such as 15-deoxy-PGJ_2_ can be attributed to both PPARγ activation and a receptor-independent mechanism, such as modulation of intracellular redox potential in osteosarcoma cells [114], but a clear contribution of CRTH2-mediated effects is also given: CRTH2 activation can induce apoptosis via autocrine stimulation of both reactive oxygen species and TNF-α production in a MAPK pathway-dependent manner in cardiomyocytes [115], and via Erk1/2 and Akt signaling in human osteoclasts [77]. Although, these in-vitro data suggest anti-tumorigenic properties, the exact role of CRTH2 in cancer is still unclear: CRTH2 expression on circulating CD4 positive cells was elevated in the late stage of non-small cell lung cancer [116], and in an experimental model using Lewis lung carcinoma cells implanted on the back of mice, CRTH2 expression was detected in vascular cells and the growing tumor [117]. Furthermore, in 277 samples of human gastric cancer, 17% of cases showed cancer cells positive for CRTH2 [118] and polarized group 2 innate lymphoid cells (ILC2) with increased levels of CRTH2 were found in the peripheral blood of gastric cancer patients [119]. These data point to a potential implication of PGD_2_ and CRTH2 in cancer, but whether beneficial or deleterious still needs to be elucidated.

## 3 Conclusion

**Fig 1:**
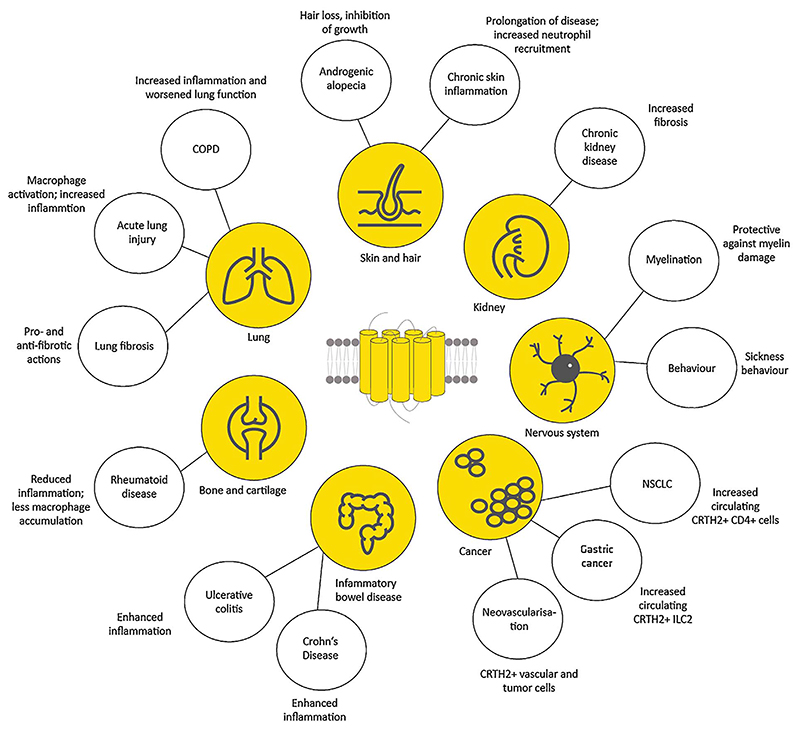
CRTH2/DP2-mediated effects beyond allergic inflammation and asthma; COPD (chronic obstructive pulmonary disease), NSCLC (non-small cell lung carcinoma).

With a plethora of actions, CRTH2-mediated effects are apparent in almost every tissue of the human body (Fig. 1). There is growing evidence that CRTH2 plays important roles in allergic inflammation of the respiratory tract and the skin; however, this does not exclude it from being a potential therapeutic target in other conditions, too. These might comprise inflammatory bowel disease, mood disturbances or even cognitive dysfunction on the one hand, and autoimmune disease such as rheumatoid arthritis, and lung and kidney fibrosis, on the other hand. In male-type baldness, CRTH2 antagonists might already be on the crossroads to becoming available for patients, soon.

## Figures and Tables

**Table 1:** Reported presence (or reactivity) and absence of CRTH2/DP2 on human and murine structural and immune cells.

	Human		
	Cell type	Reported by	Reference
CRTH2/DP2 positive	bronchial epithelium	immunostaining	[35,36]
	mast cells	immunohistochemistry, flow cytometry	[38]
	basophils	flow cytometry, mRNA expression	[39]
	eosinophils	flow cytometry, mRNA expression	[21,39]
	macrophages	flow cytometry, immunohistochemistry	[40]
	monocytes	flow cytometry, mRNA expression	[41]
	innate lymphoid cells type 2	flow cytometry	[42]
	Th2 cells	flow cytometry, mRNA expression, western blotting	[23]
	dendritic cells	flow cytometry, mRNA expression	[41]
	CD8 + T cells (positive in same cases)	flow cytometry, mRNA expression, western blotting	[23]
CRTH2/DP2 negative	Th1 cells	flow cytometry, mRNA expression	[23]
	NK cells	flow cytometry, mRNA expression	[23]
	B cells	flow cytometry, mRNA expression	[23]
	neutrophils	mRNA expression	[22,43]

	Mouse		
	Cell type	Reported by	Reference
CRTH2/DP2 positive/reactive	epithelium	reactive to CRTH2 agonism	[44]
	mast cells	mRNA expression, western blotting	[45]
	eosinophils	mRNA expression	[46]
	macrophages	mRNA expression	[47]
	monocytes	mRNA expression	[47]
	Innate lymphoid cells type 2	flow cytometry, mRNA expression	[48]
	Th2 cells	mRNA expression	[49]
	Th1 cells	mRNA expression	[49]
	CD8+ T cells	mRNA expression	[49]
	neutrophils	mRNA expression	[50]
